# Psychosocial factors at work and stress among the nursing staff of a central sterile services department

**DOI:** 10.5327/Z1679443520190453

**Published:** 2019-12-01

**Authors:** Paula Caroline Guissi, Maria Amélia Stefanutto Zihlmann Pinho, Israel Vieira, Fidelis Ranali, Daniela de Almeida Martins, Marcia Cristina das Dores Bandini, Sergio Roberto de-Lucca

**Affiliations:** 1 School of Medical Sciences, Universidade Estadual de Campinas - Campinas (SP), Brazil. Universidade Estadual de Campinas School of Medical Sciences Universidade Estadual de Campinas Brazil; 2 Occupational Health Division, Human Resources Department, Universidade Estadual de Campinas - Campinas (SP), Brazil. Universidade Estadual de Campinas Occupational Health Division Human Resources Department Universidade Estadual de Campinas Brazil

**Keywords:** nurse practitioners, hospital services, occupational stress

## Abstract

**Background:**

The international literature points out an association between stress and psychosocial factors at work (PFW) in different occupational groups. Among health care workers, providing direct patient care might be rewarding or contribute to cause stress, and central sterile services departments (CSSD) provide relevant support to in-hospital care.

**Objective:**

To investigate PFW liable to cause stress among the nursing staff of a university hospital CSSD.

**Methods:**

Quantitative and qualitative study of a non-probabilistic sample of 63 workers who responded a sociodemographic questionnaire and the Effort-Reward Imbalance (ERI) scale. Fifty-one participants were also subjected to individual interviews, which were recorded, transcribed and subjected to content analysis, resulting in thematic matrices and categories.

**Results:**

The sample mainly comprised women (92%) and was aged 45 years old, on average. Effort-reward imbalance was found for 16% of the participants. Working conditions, equipment and materials, relationship among coworkers, and management support were listed as factors related to job satisfaction or dissatisfaction. Lack of peer recognition was described as the main factor associated with dissatisfaction and stress.

**Conclusion:**

CSSD workers feel they are stigmatized and underestimated by their coworkers involved in direct patient care and the institution as a whole. Recognition of the work done is fundamental for job satisfaction and health protection and preservation.

## RESUMO |

**Introdução:**

A literatura internacional aponta a associação entre estresse e os fatores psicossociais no trabalho (FPT), que podem acometer diversas profissões. Na área da saúde, a assistência direta aos pacientes pode ser gratificante ou ter impacto no desencadeamento de estresse. Nos serviços hospitalares, a Central de Materiais Esterilizados (CME) tem importância relevante no suporte à assistência.

**Objetivo:**

Identificar os FPT com potencial de desencadear estresse ocupacional nos profissionais de enfermagem da CME de um hospital universitário.

**Método:**

Estudo quantiqualitativo de amostra não probabilística de 63 profissionais que responderam aos questionários sociodemográfico e balanço esforço-recompensa (ERI) sobre os fatores psicossociais no trabalho. Após essa etapa, foram entrevistadas 51 pessoas. As entrevistas, gravadas, transcritas e analisadas em seu conteúdo, foram organizadas em matrizes temáticas dos FPT e respectivas categorias.

**Resultados:**

A amostra identificou predominância de mulheres (92%), com idade média de 45 anos. Constatou-se desequilíbrio no ERI em 16% dos participantes. Condições de trabalho, equipamentos e materiais, relacionamentos entre colegas e suporte da gestão foram elencados como fatores de satisfação e insatisfação no trabalho. A falta de reconhecimento do trabalho pelos colegas de outros setores foi apontada como principal fator de insatisfação e estresse.

**Conclusão:**

Os profissionais da CME sentem-se estigmatizados e desprestigiados pelos colegas dos setores assistenciais e pela própria Instituição. O reconhecimento do trabalho realizado é de fundamental importância na satisfação no trabalho, proteção e preservação da saúde.

## INTRODUCTION

Psychosocial factors at work (PFW) are a result of the interrelationship among workers, the organization of work and the work environment^1^, and include: management and communication style, support from coworkers and supervisors, nature and strength of job demands, and degree of autonomy and participation of employees in decision making about the work process. PFW also concern the workers’ needs and expectations, real and symbolic recognition, career plan, and balance between work and personal life. This is thus a complex interaction with positive or negative influence on job satisfaction and the state of health of workers^2^.

Economic changes and global technological innovations in the past decades brought substantial modifications into the world of work, the structure of production, management styles, and the relationship between workers and employers, including increasing demands for productivity and individual and collective outcomes. These changes had negative consequences for organizations, as e.g. higher levels of occupational stress^3^^,^^4^.

As concerns health care, the job demands might trigger stress among workers^5^, the nursing staff in particular. One typical example is that of hospitals, in which being a member of a hierarchical multiprofessional staff might be a source of illness as a function of the degree of resilience and ability of workers to develop appropriate emotional control vis-à-vis the demands posed by patients and their families. The characteristics of hospital work and the complexity of tasks, in association with PFW, might lead to job dissatisfaction, distress and illness, manifested as sickness absenteeism, presenteeism, mental disorders and job burnout^2^^,^^6^^,^^7^^,^^8^^,^^9^.

Recent social security data indicate that mental and behavioral disorders are the third leading cause of sick leave lasting more than 15 days, being the main reason among health care workers^10^. Given the relevance of PFW for health, theoretical models and assessment instruments were developed along the past 30 years to investigate the relationship between stress and psychosocial and organizational factors^11^^,^^12^.

The Effort-Reward Imbalance (ERI) scale, developed by Siegrist in 1996 and translated for use in Brazil, is one of such instruments. Internationally acknowledged, its theoretical framework is based on the notion of perceived job dissatisfaction as the origin of occupational stress and its negative effects for the workers’ physical and mental health^13^. Job dissatisfaction is the result of imbalance between effort and commitment to work, in other words, of the workers’ belief that rewards are not proportionate to their work^14^^,^^15^.

Workers in hospital support services do not participate in direct patient care, but their almost invisible work is indispensable to ensure care provision 24/7. Most studies about PFW have focused on workers who provide direct patient care at the expense of those allocated to support services, as e.g. the central sterile services department (CSSD). CSSD is essential for surgery departments and performing specialized procedures, which occur uninterruptedly at hospitals. This population of workers is exposed to psychosocial factors associated with stress and illness as a function of ergonomic issues, inefficacious management, and lack of recognition^16^^,^^17^^,^^18^.

Given the aforementioned considerations, the aim of the present study was to investigate psychosocial and organizational factors liable to cause occupational stress among nursing professionals allocated to the CSSD of a university hospital in São Paulo, Brazil.

## METHODS

The present quantitative and qualitative study was performed at a university hospital in São Paulo from 27 August through 30 November 2018. The participants were administered questionnaires and interviewed at the CSSD during the working hours.

The analyzed CSSD is charged of the cleaning, storage and distribution of surgical materials and equipment used at the various hospital departments. Decontamination involves four steps:

- Reception and initial cleaning and disinfection;
- Organization and distribution of clean and dry materials according to use, medical specialty and procedures;
- Sterilization;
- Storage.

Access to the CSSD is restricted to the staff, which is expected to meet continuous demands in terms of volume, in addition to peak demands. Tasks involve standardized high-technology procedures, which in addition to manual work also require cognitive skills to ensure high quality to the entire process^15^. At the analyzed CSSD workers are distributed across shifts, including rotations between workstations according to their technical skills and/or medical restrictions.

Based on a list provided by the CSSD management we located 70 workers who met the inclusion criteria: having worked more than 6 months at the CSSD, not currently on leave, and providing informed consent. The exclusion criteria were: refusal to provide informed consent and returning questionnaires with missing data. In the first (quantitative) phase of the study we administered a questionnaire with sociodemographic and occupational data: age, sex, length in the job, position, employment relationship, working time, reason to work at CSSD, second job, and weekly working hours.

Next we administered the short version of ERI cross-culturally adapted for use in Brazil by Silva and Barreto^19^. This questionnaire comprises 23 items responded on a Likert scale and distributed across three domains:

- Physical and cognitive effort at work (6 items);
- Subjective reward for effort (11 items);
- Commitment to work (6 items).

The results are assessed by means of the equation E/(R*i), where E represents the score on the effort domain, R the score on the reward domain, and i a factor of correction calculated by dividing the score on effort by the score on reward. Imbalance is defined as a score >1^13^.

The quantitative data were organized, tabulated and analyzed using software Microsoft Excel.

In the second phase of the study we invited all the participants to in-depth interviews based on five trigger questions:

- Do you like working at the CSSD?;
- What does working at the CSSD mean to you?;
- Please, mention positive aspects and difficulties of work at the Clinical Hospital CSSD;
- What would you like to change at the CSSD?;
- What would you like to change in CSSD work?

The interviews lasted about 25 minutes and were recorded and transcribed following Bardin’s methods^20^. We first performed an unfocused reading of the transcripts to establish their content and identify representative thematic matrices and categories previously validated by the participants.

The present study was approved by the ethics committee of Universidade Estadual de Campinas, ruling no. 2,610,876. The informed consent form signed ensures confidentiality and anonymity of the collected data.

## RESULTS

Table 1 describes the participants’ sociodemographic and occupational data. Seventy workers were eligible as per the inclusion criteria, but six were excluded because they responded the questionnaires wrongly, and one further subject refused participation. The final sample therefore included 63 participants.

**Table 1.** Occupational variables. Campinas, Brazil, 2019 (n=63).

Variables	N	%
Position		
Nurse	9	14.3
Nursing technician	54	85.7
Shift		
Administrative	4	6.3
First	23	36.5
Second	18	28.6
Third	18	28.6
Reason to work at CSSD		
Direct hiring	33	52.4
Requested transferal	20	31.7
Medical restrictions	10	15.9
Second job		
Yes	6	9.5
No	57	90.5
Years of work at CSSD		
Up to 5	33	52.4
6 to 10	14	22.2
11 to 20	9	14.3
More than 20	7	11.1
Total	63	100

CSSD: Central Sterile Services Department.

Most of the participants were female (92.1%), 70% were aged 41 to 60, which indicates a rather old workforce. Most of the participants worked 30 to 35 hours/week and only 9.5% reported to have a second job.

About 52.4% of the sample had been directly hired to the CSSD, while all the others had been transferred from another department upon their own request or due to medical restrictions (15.9%). The participants’ perceived job satisfaction as assessed by ERI is described in Table 2.

**Table 2.** Job satisfaction according to Effort-Reward Imbalance (ERI), Campinas, Brazil, 2019 (n=63).

Variables	N	%
ERI results		
ER balance	53	84.1
ER imbalance	10	15.9
Total	63	100

Considering effort, the job demands and institutional reward, most participants (84.1%) were categorized as in state of balance. One single participant exhibited an overcommitment as cause of imbalance. Eight participants (12.7%) were categorized as borderline (>0.8).

Fifty-one participants agreed to be interviewed. The narratives were subjected to unfocused reading and selection of representative thematic matrices and categories for analysis, which are described in Table 3. Next we illustrate some representative transcripts of each identified category:

• Self-perception of CSSD
- *“I love what I do”* (I34).
- *“As they say, it’s the heart of the institution, it really is”* (I15).
- *“The hospital can’t work without it”* (I13).
• Institutional perception
- *“They don’t understand our job’s complexity”* (I34).
- *“They believe we just clean pots and pans”* (I21).
- *“There’s too much prejudice”* (I4).
- *“I used to believe one didn’t have to be too much intelligent […] that even the cleaning ladies could do this job”* (I13).
• Equipment and materials
- *“There aren’t machines to clean the materials right”* (I28).
- *“The equipment requires better maintenance, because one has this feeling the equipment only becomes visible when it’s broken”* (I16).
- *“Our materials and surgical kits are incomplete, there aren’t replacements”* (I34).
• Working conditions
- *“The reception section needs some reform”* (I22).
- *“The cupboards to store sterilized materials are not enough”* (I18).
- *“The cupboards aren’t duly labeled, and workers may make mistakes because they are overloaded”* (I23).
• Relationship among coworkers
- *“Some guys are really committed to what they do, but others are totally careless”* (I35).
- *“There’s this relationship among the nurses, too much power struggle”* (I33).
- *“Some guys because they’re friends with supervisors believe they have more rights and privilege”* (I14).
• Adequacy of human resources
- *“There’s a shortage of workers, on occasions, depending on the day of the week and the work schedule, one has to accomplish three tasks at the same time”* (I32).
- *“More workers are needed. You got a medical certificate and have some restriction? Come to the CSSD”* (I25).
• Technical development and training
- *“Here one needs a lot of knowledge, it has a lot of potential”* (I17).
- *“Here you have to have a lot of knowledge and keep learning more”* (I22).
- *“Continuing education would be necessary”* (I18).
• Management and supervisors’ support
- *“There’s a lack of understanding with the supervisors, if it’s not OK, nothing else is”* (I10).
- *“Management needs to improve […] The managers’ responsibility”* (I25).
- *“One word they’d say to us would be enough. They don’t need to send us letters”* (I13).

**Table 3.** Thematic matrices and categories, Campinas, Brazil, 2019 (n=51).

Thematic matrices	Categories
Job meaning and satisfaction	Institutional perception
	Self-perception of CSSD
Working conditions, materials and equipment	Working conditions
	Materials and equipment
Psychosocial factors at work	Relationship among coworkers
	Development/training
	Adequacy of human resources
	Management and supervisors’ support

CSSD: Central Sterile Services Department.

## DISCUSSION

The analyzed staff profile is quite peculiar by comparison to that reported in other studies. Only 9.5% of these workers had a second job, which was described as a differential and positive characteristic of the institution. In a study performed at a hospital in the interior of the state of São Paulo^7^^,^^21^, 25% of the nursing staff reported having a second job as a function of a comparatively lower salary.

Since the analyzed institution is a public hospital with competitive class employees, about 70% of the staff was aged over 40. Besides aging, 16% of the employees were allocated to the CSSD due to medical restrictions (derived from musculoskeletal and mental disorders)^18^. This situation may result in work overload for the younger workers.

About 80% of the staff was directly hired or requested a transfer to the CSSD. This finding indicates that exemption from direct patient care might be a motivational factor with positive impact on the involved workers.

The results obtained from ERI and the interviews reinforce the participants’ perceived job satisfaction. For instance, as one interviewee stated “*The hospital can’t work without it* [CSSD].” These workers are able to find themselves in their job, because they consider the CSSD essential to the hospital’s good functioning. Subjective recognition of one’s own work - “*I love what I do*” - is a protective factor against illness. Although they resent their supervisors’ lack of appreciation - ”*One word they’d say to us would be enough. They don’t need to send us letters” -* the participants’ narratives evidenced the relevance of the CSSD and its impact on the quality of care delivery, even if only indirectly^18^.

Teamwork and coworker support might contribute to make work pleasurable, because workers can share their experiences and thus attenuate distressing factors in the workplace^13^. However, the narratives - ”*Some guys are really committed to what they do, but others are totally careless” -* also evidenced problems and conflict with coworkers and supervisors, because “*there is too much power struggle,”* which compels some workers to adopt a competitive attitude. The latter can be seen as a personal coping strategy - ”*Some guys because they’re friends with supervisors believe they have more rights and privilege” -* that might interfere with teamwork and the professional growth of the group^22^.

The narratives pointed out also other PFW which contributed to the participants’ self-perceived occupational stress: fast pace of work and high productivity demands, made worse by absenteeism, working in a closed environment without communication with other hospital departments, the prejudiced or stereotypic views of other hospital workers about the role of CSSD employees^23^ - ”*They believe we just clean pots and pans” -* and the transferal of workers with medical restrictions.

Poor working conditions, as e.g. inadequate equipment maintenance - manifested in narratives such as “*There aren’t machines to clean the materials right”* and “*The cupboards to store sterilized materials are not enough”* - were emphasized by the participants as factors which interfered with the appropriate performance of their tasks^24^.

The participants further expressed a need for training and updating, since they acknowledged that “*Here one needs a lot of knowledge, it has a lot of potential.”* They also observed they can contribute to the continuous improvement of a department essential to the hospital’s good functioning.

While we cannot assert that working at CSSD is protective against occupational stress, the employees’ reported experience points to the relevance of investigating hospital support services not involved in direct patient care.

As limitations of the present study, the sample did not include all the CSSD employees. Then, specific aspects of the analyzed institution and the participants’ sociodemographic and occupational profile preclude generalizing the results to the CSSD and other support services of other hospitals.

## CONCLUSION

The results of the present study point to the relevance of investigating employees allocated to hospital support services, who by not being involved in direct patient care are often invisible within the institution.

High job demands, made worse by absenteeism, problems in interpersonal relationships, lack of supervisors’ support and prejudice, and underestimation of hospital workers not involved in direct patent care were the main PFW reported by the participants. Nursing professionals reported to feel diminished in the eyes of coworkers who provide direct patient care, as well as of the institution as a whole. However, we could observe they were satisfied with their job, and also the relevance of the symbolic recognition of their occupational role as an essential aspect of job satisfaction.

The results of the present study might contribute to ground health promotion actions, as well as to the acknowledgment of the strategic relevance of CSSD and other hospital support services.
